# Supplementary material for: GluA3 subunits are required for appropriate assembly of AMPAR GluA2 and GluA4 subunits on cochlear afferent synapses and for presynaptic ribbon modiolar–pillar morphology
Source: eLife. 2023 Jan 17;12:e80950. doi: 10.7554/eLife.80950 (PMC9891727; doi:10.7554/eLife.80950)
Supplement: Figure 1—source data 2. [file elife-80950-fig1-data2.zip › Figure 1-source data 2/Figure 1-data2 - Raw gels_Gria2 Gria4 flip and flop-1 copy.pdf]

PCR gels

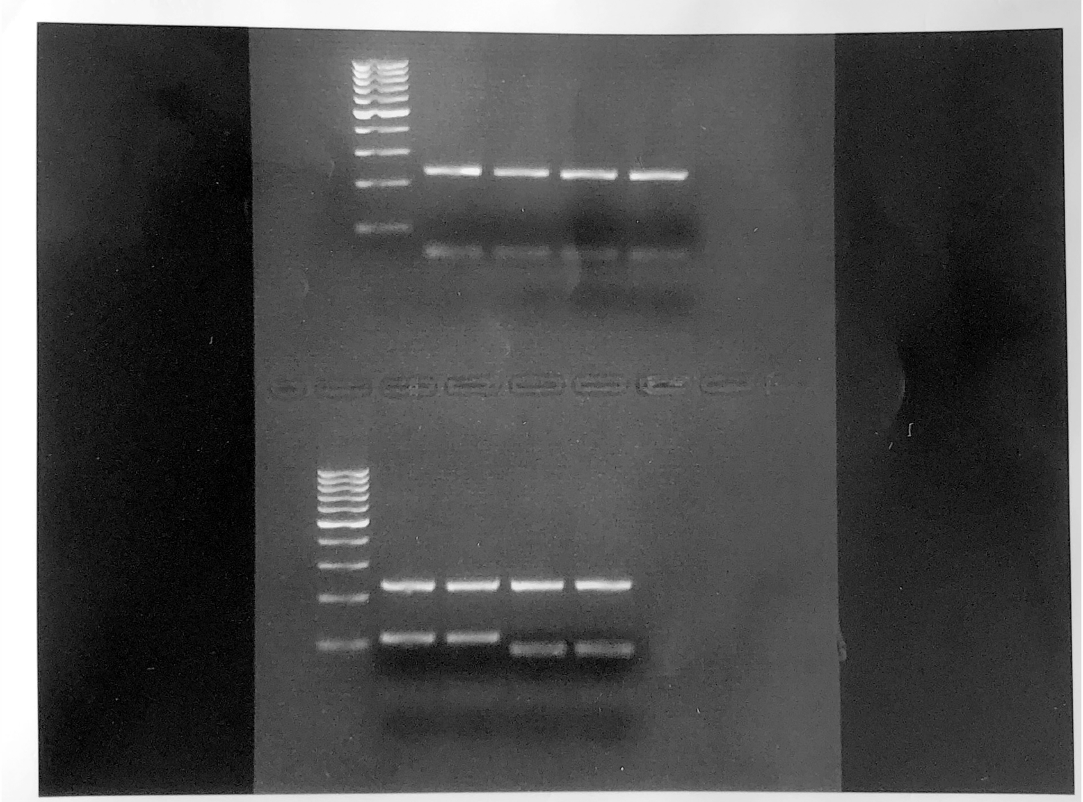

24-May-2018 11:54:43 Low=0 High=4095 Gamma=1.0 Exposure = 0.500 secs

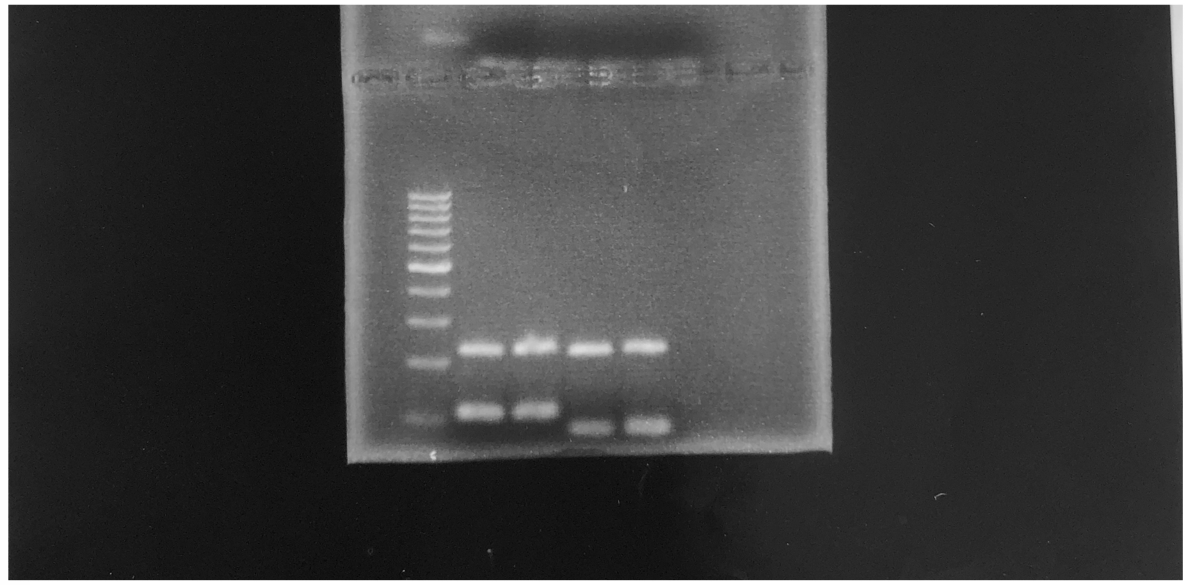

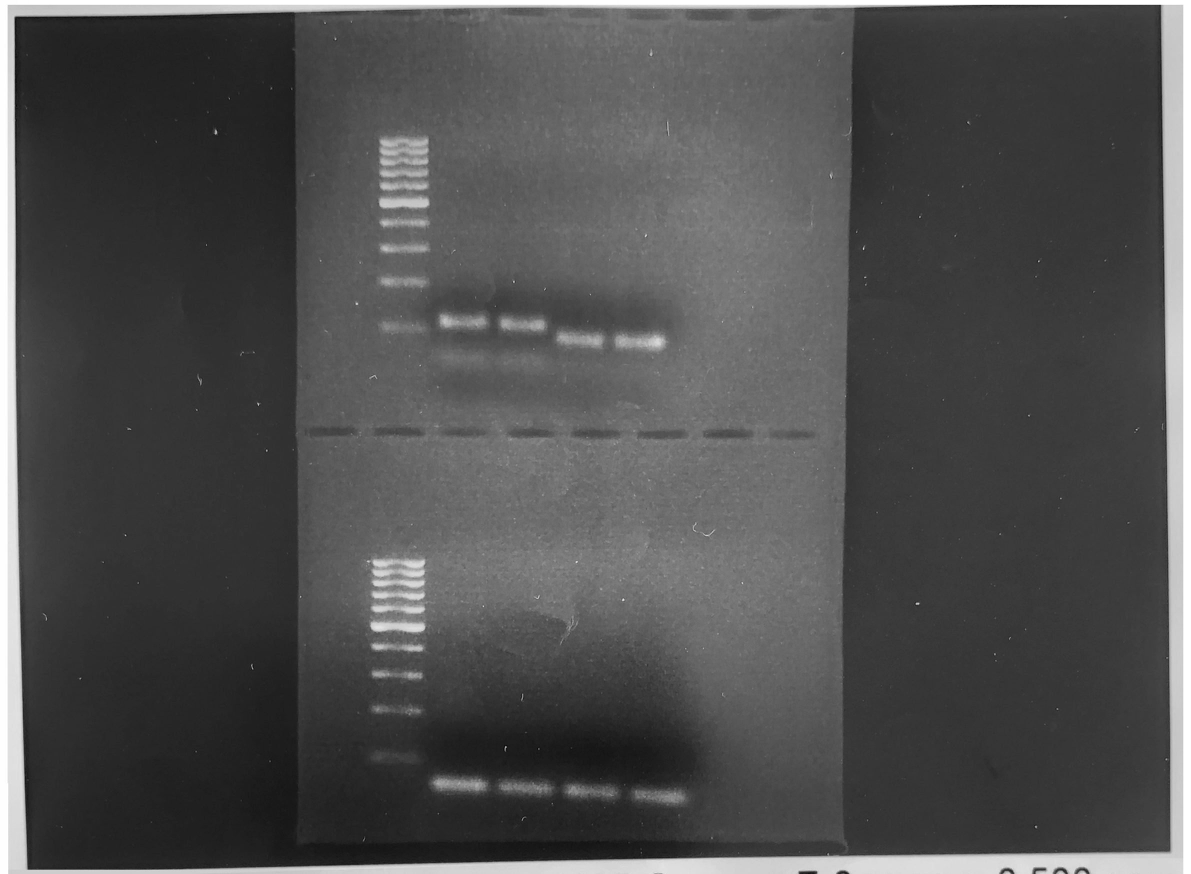

25-May-2018 12:40:11 Low=0 High=4095 Gamma=1.0 Exposure = 0.500 secs

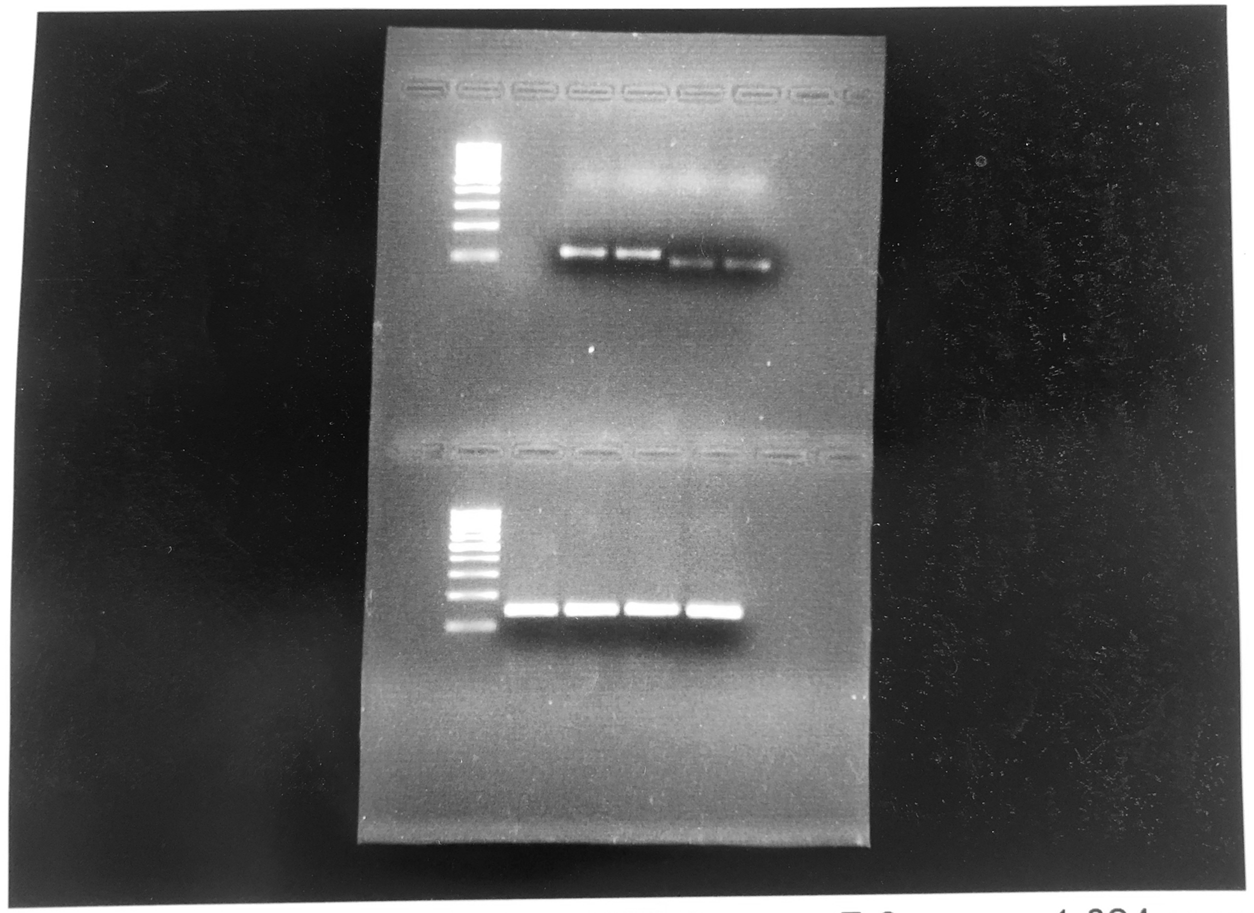

12-Jun-2018 16:53:03 Low=0 High=4095 Gamma=1.0 Exposure = 1.024 secs

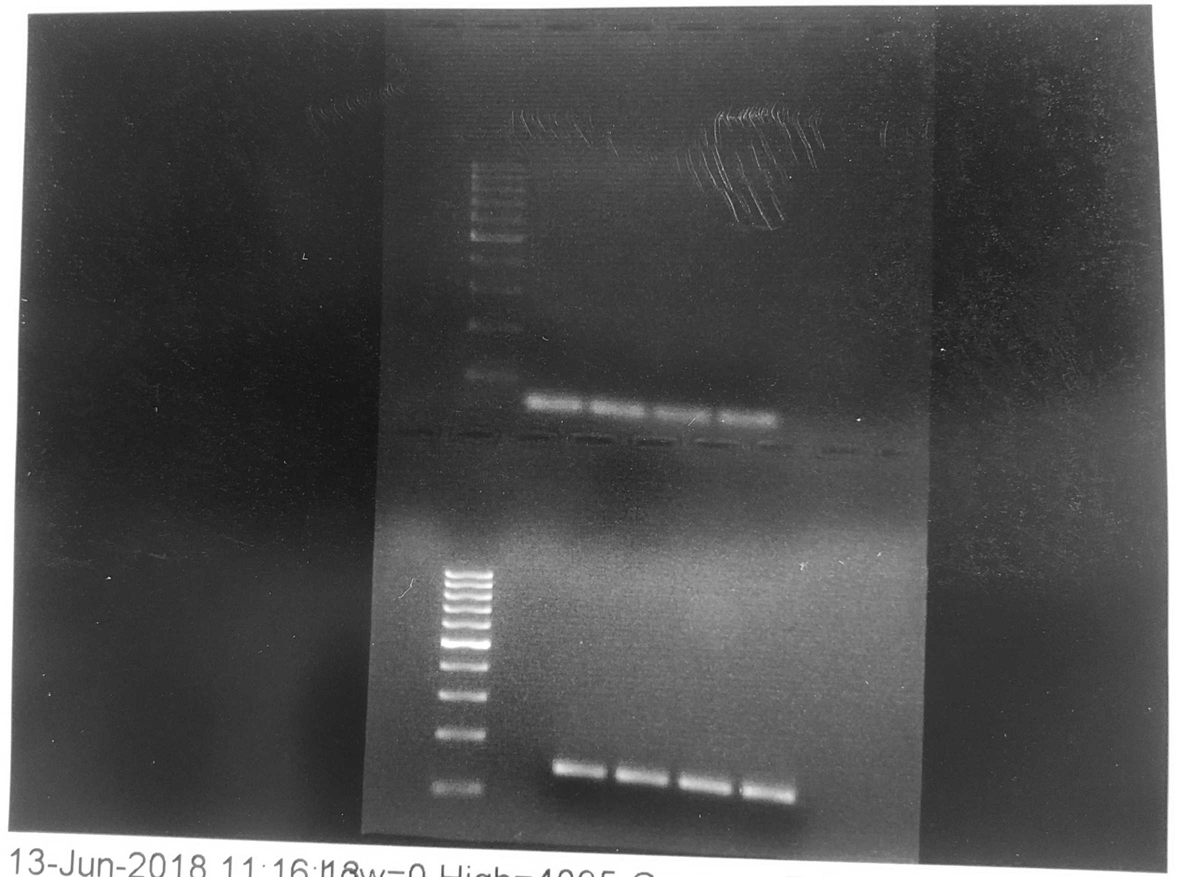

13-Jun-2018 11:16:13 Low=0 High=4095 Gamma=1.0 Exposure = 1.024 secs
