# Supplementary figures and images for: GluA3 subunits are required for appropriate assembly of AMPAR GluA2 and GluA4 subunits on cochlear afferent synapses and for presynaptic ribbon modiolar–pillar morphology
Source: eLife. 2023 Jan 17;12:e80950. doi: 10.7554/eLife.80950 (PMC9891727; doi:10.7554/eLife.80950)

PCR gels

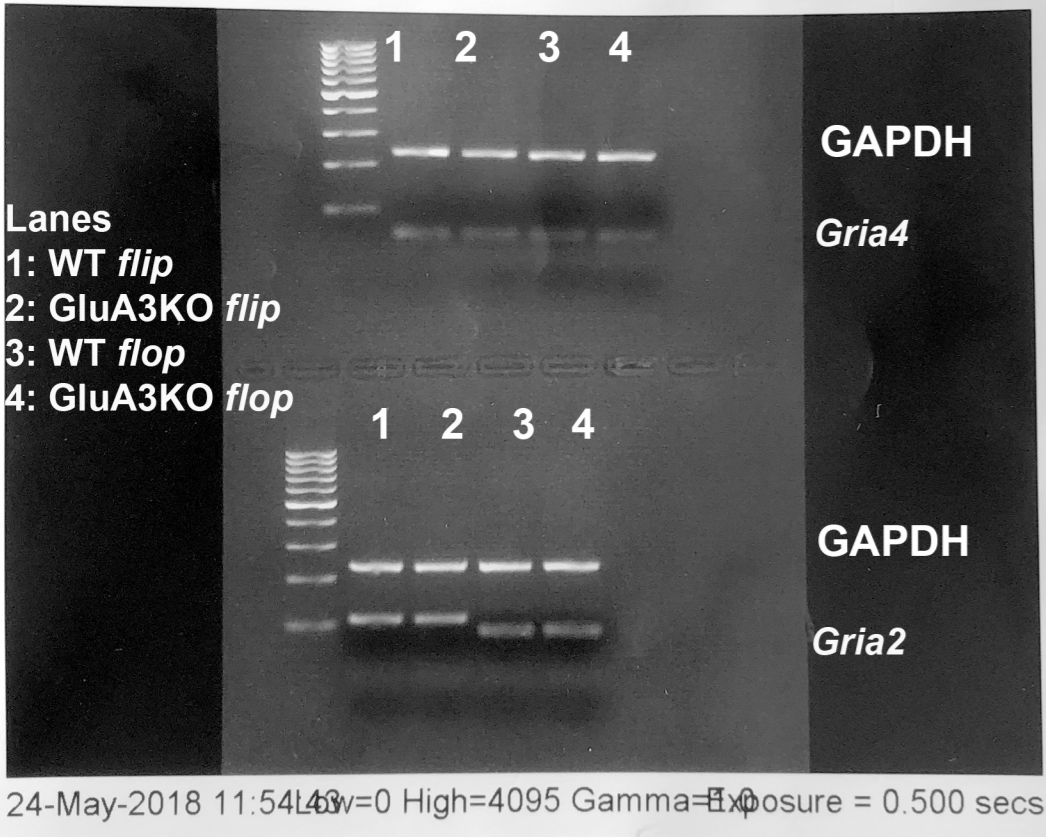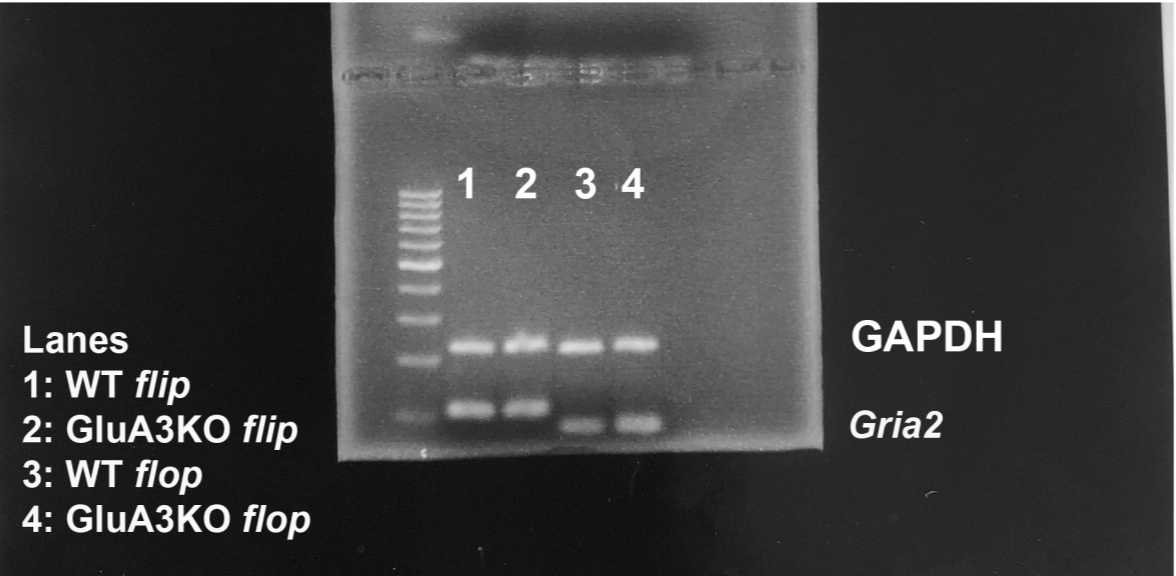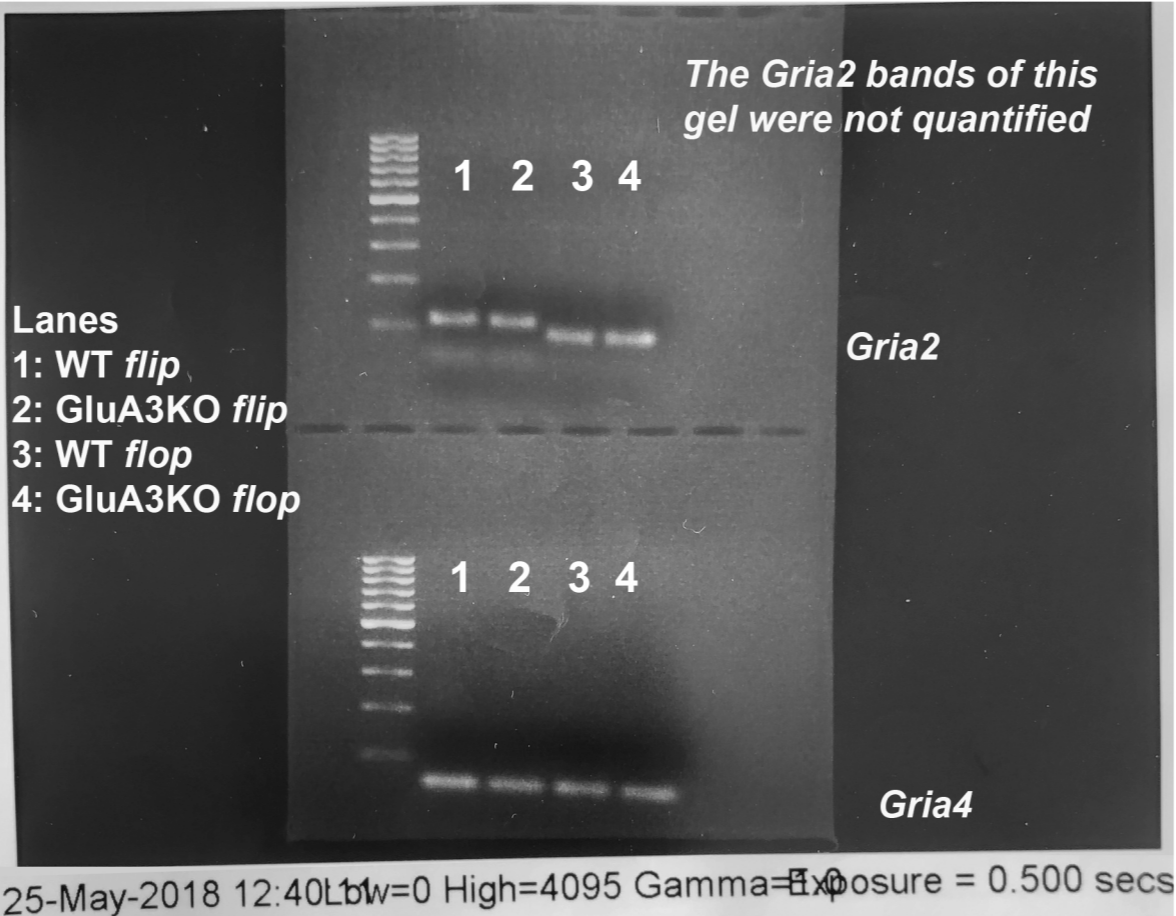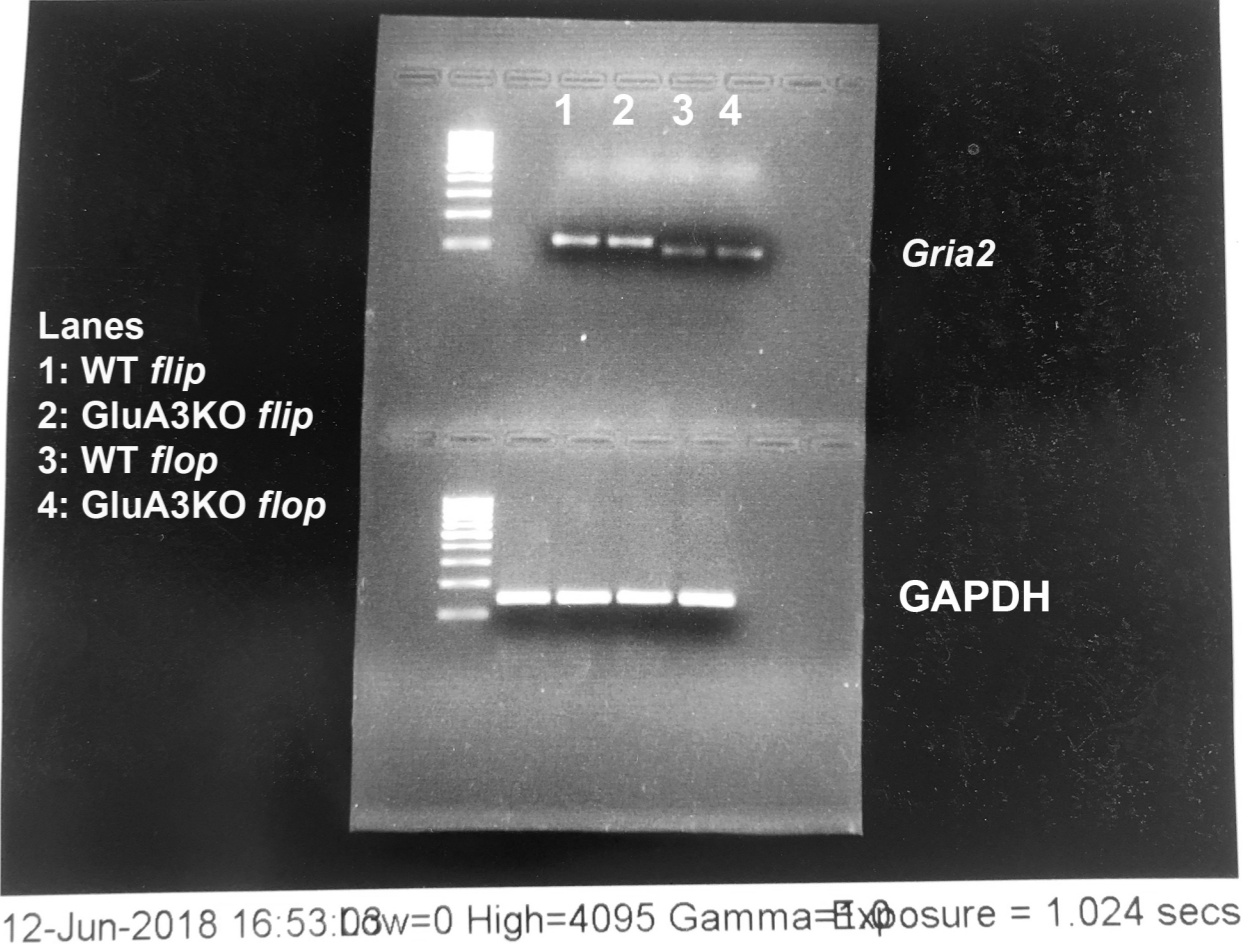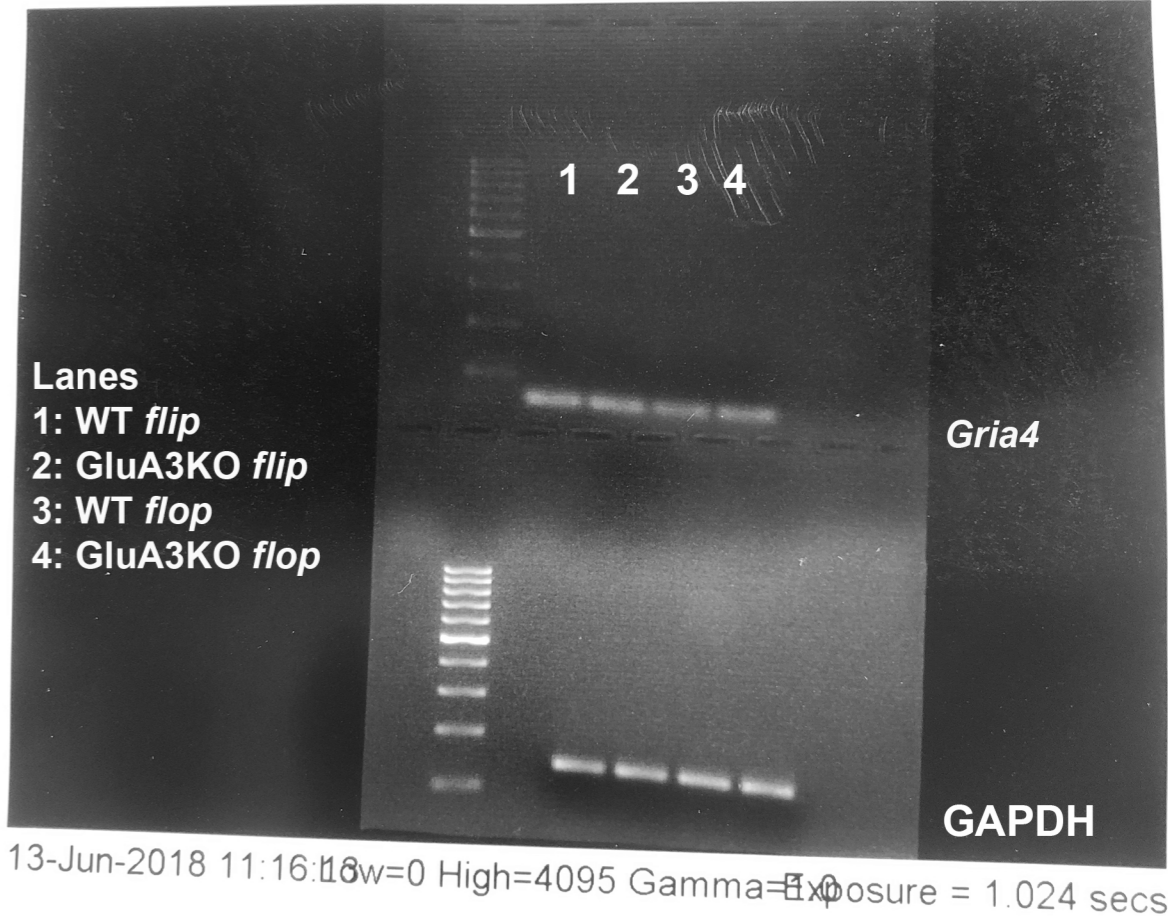

Supplement: Figure 1—source data 3. [file elife-80950-fig1-data3.zip › Figure 1-source data 3/Figure 1-data3 Fig1-data3 - Labelled gels, Gria2 Gria4 flip and flop copy.pdf]
